# Phase angle is a useful bioelectrical marker for skeletal muscle quantity and quality in hospitalized elderly patients

**DOI:** 10.1097/MD.0000000000031646

**Published:** 2022-11-11

**Authors:** Jiaxu Geng, Yanan Wei, Qian Xue, Lihua Deng, Jingtong Wang

**Affiliations:** a Department of Gerontology, Peking University People’s Hospital, Beijing, China.

**Keywords:** computed tomography, phase angle, sarcopenia, skeletal muscle area index, skeletal muscle density

## Abstract

To analyze whether phase angle (PhA) can be a useful bioelectrical marker for skeletal muscle quantity and quality in hospitalized elderly patients. Two hundred hospitalized elderly patients were included in this retrospective observational study. PhA was obtained by Bioelectrical Impedance Analysis, skeletal muscle area index (SMI) and skeletal muscle density (SMD) were measured at the third lumbar vertebra level in computed tomography images using SliceOmatic software. PhA was positively associated with SMD and SMI, with correlation coefficients of 0.629 and 0.674, respectively. Multiple logistic regression analysis showed that 1° reduction of PhA was significantly associated with low SMI [odds ratio (OR) = 4.331 (1.681–11.161)] and low SMD [OR = 6.418 (2.963–13.899)]. Receiver operating characteristic curve analysis showed that the area under the curve (AUC) for PhA to identify patients with low SMI was 0.772 for male and 0.784 for female; the AUC for PhA to identify low SMD patients was 0.829 for male and 0.812 for female; the AUC for PhA to identify low SMD combined with low SMD patients was 0.801 for male and 0.773 for female. The results of this study showed that PhA was highly related to SMI, which can indicate the quantity of skeletal muscle in the entire body, and was highly related to SMD, which can be used to assess skeletal muscle quality. Therefore, PhA may be a useful bioelectrical marker for skeletal muscle quantity and quality.

## 1. Introduction

Aging is associated with a loss of skeletal muscle quantity/mass and strength, but a decrease in muscle strength occurs faster than skeletal muscle quantity.^[1,2]^ A 3-years longitudinal study of 1800 healthy older adults found that lean body mass decreased by approximately 1% per year, but muscle strength decreased by up to 4% during the same period.^[3]^ Loss of muscle quantity explains only part of the loss of muscle strength, because skeletal muscle quantity loss is accompanied by changes in skeletal muscle quality, such as skeletal muscle fat infiltration. Skeletal muscle fat infiltration is closely related to the decline in skeletal muscle strength and physical activity and is independent of skeletal muscle quantity.^[4–6]^

Skeletal muscle fat infiltration can be quantified by muscle biopsy or noninvasive analysis such as computer tomography (CT), magnetic resonance imaging (MRI), and ultrasound. Muscle biopsy is the gold standard; however, it is an invasive procedure that limits its application. MRI is accurate, but it is expensive and not portable, and some patients with metal implants or who are claustrophobic cannot undergo MRI examinations. The echo intensity obtained from ultrasound images is also used to assess skeletal muscle composition, and ultrasound has no risk of ionizing radiation, which is convenient and portable; however, the replicability of the results is questioned considering the differences in measurements between examining physicians. There is no accepted diagnostic cutoff value, which is still in the research stage.^[7]^

Previous studies have shown that the The third lumbar (L3) level skeletal muscle area derived from CT is highly correlated with total body skeletal muscle mass; therefore, CT has become a recognized method for measuring skeletal muscle quantity.^[8–11]^ In addition, CT is also one of the most widely used imaging tools for evaluating skeletal muscle quality by assessing skeletal muscle fat infiltration. According to the principle of CT imaging, the density of skeletal muscle is higher than that of fat, lower skeletal muscle density meaning higher degree of skeletal muscle fat infiltration. Meanwhile, skeletal muscle fat infiltration measured by CT is highly correlated with that measured directly by biopsy.^[12,13]^ Therefore, CT can simultaneously assess skeletal muscle quantity and quality. However, CT also has the disadvantages of ionizing radiation, is not easy to carry, and is expensive.

Bioelectrical Impedance Analysis (BIA) is widely used for body composition analysis. However, BIA relies on the estimation formula of specific populations to calculate skeletal muscle mass, and is easily affected by the hydration status of the patient. Therefore, the effectiveness and accuracy of BIA has been questioned.^[14]^ The phase angle is a raw measurement of the BIA. The main raw measurements of the BIA are resistance, reactance, impedance and phase angle. Resistance (R) corresponds to the impedance component of the current flowing through the liquid, whereas Reactance (Xc) corresponds to the impedance component of the current flowing through the cell membrane. A healthy cell membrane acts as a good capacitor that stores charge and causes current delay. Reactance reflects the energy storage capacity of the cell membrane and is positively correlated with cell number and membrane integrity. The formula Phase angle (PhA) =-Arctang (Xc/R) × 180 × π is used to calculate the PhA from the resistance and reactance. PhA is positively correlated with reactance and negatively correlated with resistance.^[15,16]^

Higher PhA values indicate greater cell mass and better membrane integrity, whereas lower PhA values indicate cell death or diminished membrane integrity.^[15,16]^ PhA can avoid errors caused by the equation estimation itself, and there is no need to assume a constant hydration state.^[17]^ Considering that PhA is a marker of cell function and cell mass, PhA may be able to simultaneously assess skeletal muscle quantity and quality. A study of colon cancer patients in Brazil showed that PhA was positively associated with CT-measured skeletal muscle area index and skeletal muscle density, and PhA had a good predictive ability for identifying low Skeletal muscle area index (SMI) and Skeletal muscle density (SMD) measured by CT.^[18]^

PhA is a simple and noninvasive bioelectrical marker. Based on this marker, we can connect the basic and clinical sciences. The techniques generated by basic science can be translated into new approaches for the monitoring of skeletal muscle atrophy.^[19,20]^

PhA can assess the body composition of outpatient, inpatient, and community patients, whereas CT has disadvantages such as ionizing radiation, high cost, and difficulty in large-scale application. The purpose of this study was to explore the value of PhA in assessing skeletal muscle quantity and quality by comparing it with the gold standard method of CT.

## 2. Methods

### 2.1. Participants

This study was performed in the geriatric department of our hospital. Patients aged ≥ 60 years who completed both BIA and abdominal CT examinations between August 2018 and October 2020 were included. The exclusion criteria were as follows: patients with artificial implants or pacemaker implantation not suitable for the measurement of BIA; patients with metal implants in the second to fourth lumbar vertebrae (*L*2–*L*4) level or insufficient image quality, which may affect the measurement of skeletal muscle parameters; patients with unstable body composition or hydration status, such as dialysis, heart failure, severe edema, hormone use, hyperthyroidism, and hypothyroidism; BIA and CT measurement time space > 14 days. The study was approved by the ethics committee of Peking University People’s Hospital (approval number NO.2021PHB183-001). All data were obtained retrospectively, and informed consent was not required.

### 2.2. Bioelectrical impedance analysis

Body composition was assessed using a BIA device (In Body 770, Biospace company). Patients were asked to fast for 2 hours, empty their bladders, and wear thin clothes. During the test, the patient stood barefooted on 2 metal electrodes, holding the metal electrode with their 2 thumbs and naturally straightening their arms at an angle of 15° until the end of the test. After the test, the PhA value was recorded at a frequency of 50 kHz.

### 2.3. Computed tomography scan measurements

Unenhanced abdominal CTs were performed for physical examinations. Each CT image was a 1-mm slice. The CT images at the L3 level were selected for measurement of the skeletal muscle area (SMA), and then normalized to CT-SMI (SMA/height^2^, cm^2^/m^2^). Based on previous reports, we discriminated skeletal muscle tissue using Hounsfield Unit (HU) thresholds between -29 and + 150.^[21]^ SMD at the L3 level was also measured. Low CT-SMI was defined as less than 40.3 cm^2^/m^2^ and 30.8 cm^2^/m^2^ for males and females, respectively.^[22]^ Low CT-SMD was defined as less than 35.5 HU and 32.5 HU for males and females, respectively.^[23]^ A trained researcher measured SMA and SMD applying the SliceOmatic v5.0 software (TomoVision, Canada) on a slice of L3 showing both transverse processes. All CT files were anonymized to avoid bias caused by subjective factors of the researchers. 50 CT images were selected randomly, SMA and SMD were measured by 2 experienced evaluators. The intraclass correlation coefficients for interobserver and intraobserver reliabilities were ≥ 0.980.

### 2.4. Statistical analysis

Continuous and categorical values were expressed as mean ± SD and number (percentage), respectively. Continuous variables were compared using the Mann–Whitney U test or Student’s *t*-test, whereas the chi-squared test was used to compare categorical variables. The correlation between PhA and SMI, meanwhile correlation between PhA and SMD was analyzed by Pearson correlation and logistic regression analysis. Receiver operating characteristic curves were constructed to analyze the ability of PhA to identify low SMI, low SMD, and low SMI combined with low SMD. The data were analyzed using IBM SPSS version 26. *P* < .05 means that the difference was statistically significant.

## 3. Results

### 3.1. Study population

Between August 2018 and October 2020, 239 hospitalized patients underwent both BIA and CT examinations. 39 patients were excluded for the following reasons: a time gap of more than 14 days between CT and BIA (n = 2), the presence of metal artifacts at the *L*2–*L*4 level, which may affect SMD measurement (n = 10), hyperthyroidism or hypothyroidism (n = 4), and age < 60 years (n = 23). Finally, 200 patients were included. The average interval between BIA and CT assessment was 3.6 ± 3.0 days. Patient characteristics classified by sex are shown in Table 1. The mean age of the patients in the study was 74.68 ± 8.37 years old, and 122 (61%) were male. There were no statistically differences in age, albumin (Alb), blood urea nitrogen, estimated glomerular filtration rate, Glycosylated hemoglobin_,_ systolic pressure, hypertension, diabetes and cardiovascular disease between male and female patients (*P* > .05). The male group showed higher height, weight, Body mass index (BMI), PhA, SMD, SMI, and Hemoglobin (Hb) level than the female group (*P* < .05).

**Table 1:** Characteristics of participants (n = 200).

	All	Male	Female	*p*
	(n = 200)	(n = 122)	(n = 78)	
Age (yr)	74.68 ± 8.37	75.39 ± 8.70	73.56 ± 7.73	.109
Weight (kg)	67.78 ± 12.21	72.77 ± 10.40	59.98 ± 10.70	<.001
Height (cm)	164.44 ± 7.94	169.08 ± 5.85	157.18 ± 4.65	<.001
BMI (kg/m^2^)	24.99 ± 3.73	25.44 ± 3.29	24.28 ± 4.27	.033
Hb (g/L)	131.34 ± 15.28	136.18 ± 14.60	123.76 ± 13.15	<.001
Alb (g/L)	38.51 ± 3.59	38.44 ± 3.29	38.60 ± 4.04	.757
BUN (mmol/L)	5.79 ± 2.07	6.00 ± 2.22	5.45 ± 1.76	.064
eGFR (mL/(min·1.73 m^2^))	78.04 ± 17.21	77.65 ± 17.11	78.67 ± 17.45	.682
HbA1c (%)	6.48 ± 1.31	6.53 ± 1.35	6.41 ± 1.24	.556
Systolic pressure (mm Hg)	131.02 ± 13.98	131.06 ± 14.40	130.95 ± 13.37	.957
Hypertension, n (%)	138 (69.0)	87 (71.3)	51 (65.3)	.377
Diabetes, n (%)	79 (39.5)	49 (40.2)	30 (38.5)	.810
Cardiovascular disease, n (%)	51 (25.5)	36 (29.5)	15 (19.2)	.104
PhA (°)	4.43 ± 0.73	4.66 ± 0.69	4.05 ± 0.62	<.001
SMD (HU)	30.90 ± 7.20	33.37 ± 6.15	27.05 ± 7.07	<.001
SMA (cm^2^)	111.53 ± 27.89	127.54 ± 21.57	86.50 ± 15.46	<.001
SMI (cm^2^/m^2^)	40.89 ± 8.31	44.64 ± 7.30	35.02 ± 6.13	<.001

Alb = albumin, BMI = body mass index, BUN = blood urea nitrogen, eGFR = estimated glomerular filtration rate, Hb = hemoglobin, HbA1c = Glycosylated hemoglobin, HU = Hounsfield units, PhA = phase angle, SMA = skeletal muscle area, SMD = skeletal muscle density, SMI = skeletal muscle area index.

### 3.2. Comparison of phase angles of different skeletal muscle components

**Figure 1.**
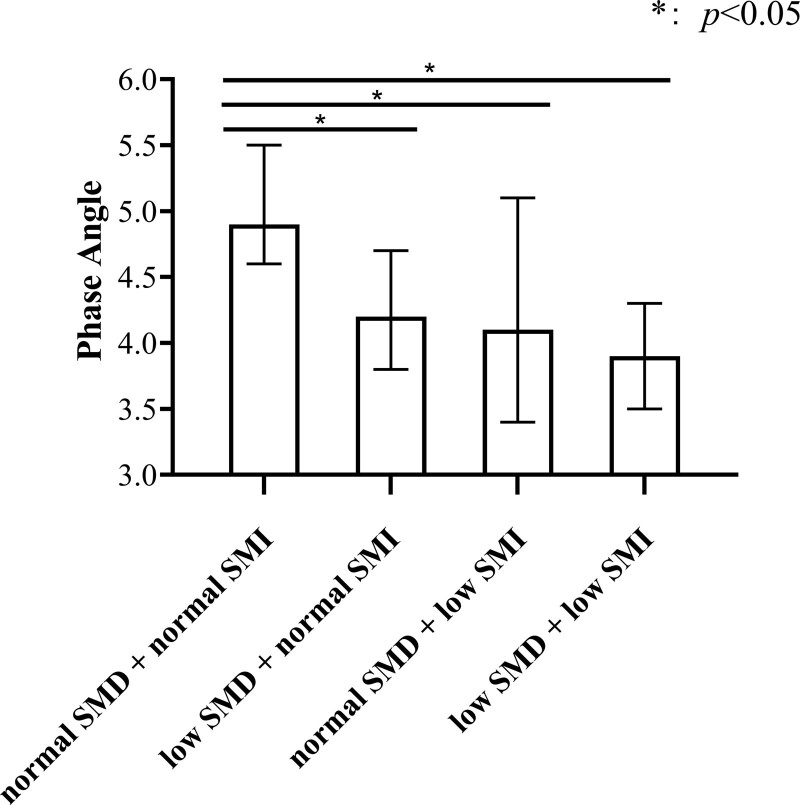
Comparison of phase angle of different skeletal muscle components. SMI = skeletal muscle area index, SMD = skeletal muscle density.

Phase angle of patients with normal SMD normal + SMI (n = 59), low SMD + normal SMI (n = 85), normal SMD + low SMI (n = 5), and low SMD + low SMI (n = 51) were compared. The results are presented as M (P25, P75), normal SMD + normal SMI = 4.9° (4.6°, 5.5°); low SMD + normal SMI = 4.2° (3.8°, 4.7°); normal SMD + low SMI = 4.1° (3.4°, 5.1°); low SMD + low SMI = 3.90° (3.5°, 4.3°). As shown in Figure 1, compared with the PhA of normal SMD + normal SMI patients, PhA of low SMD + normal SMI, normal SMD + low SMI, and low SMD + low SMI patients were lower (*P* < .05).

### 3.3. Correlation between pha and SMI and SMD

Pearson correlation analysis showed that PhA was positively associated with SMD and SMI, with correlation coefficients of 0.629 and 0.674, respectively. Both SMI and SMD were negatively correlated with age (*P* < .001). SMI was positively correlated with BMI (*P* < .001), whereas SMD and BMI were not significantly correlated, as shown in Table 2.

**Table 2:** Correlation of SMD and SMI with PhA, sex, age, and BMI.

	SMI (cm^2^/m^2^)		SMD (HU)	
	*r*	*p*	*r*	*p*
Sex	0.595	<.001	0.413	<.001
Age	–0.286	<.001	–0.355	<.001
BMI	0.615	<.001	–0.073	.303
PhA	0.674	<.001	0.629	<.001

BMI = body mass index, HU = Hounsfield units, PhA = phase angle, SMD = skeletal muscle density, SMI = skeletal muscle area index.

Logistic regression was used to analyze the relationship between low SMD, low SMI and PhA. The results showed that 1° reduction of Phase angle was significantly related to low SMI [odds ratio = 4.331, 95% confidence interval (95%CI): 1.681–11.161], and low SMD (odds ratio = 6.418, 95%CI: 2.963–13.899), as shown in Table 3.

**Table 3:** Multiple logistic regression analysis between low SMI, low SMD and PhA.

PhA	Low SMI (cm^2^/m^2^) (n = 56)			Low SMD (HU) (n = 136)		
	OR	95%CI	*p*	OR	95%CI	*p*
Crude	3.701	2.155–6.358	<.001	8.127	4.333–15.244	<.001
Adjusted	4.331^a^	1.681–11.161	.002	6.418^b^	2.963–13.899	<.001

95%CI = 95% confidence interval, BMI = body mass index, HU = Hounsfield units, OR = odds ratio, PhA = phase angle, SMI = skeletal muscle area index, SMD = skeletal muscle density.

a Adjusted for age, sex and BMI.

b Adjusted for age and sex.

### 3.4. The ability of pha to identify low SMI and low SMD

Receiver operating characteristic curve analysis showed the ability of PhA to identify low SMI, low SMD, and low SMI combined with low SMD. The Area under curve (AUC) for PhA to identify low SMI patients was 0.772 (95%CI: 0.684–0.860, *P* < .01) for male and 0.784 (95%CI: 0.672–0.895, *P* < .01) for female, AUC for PhA to identify low SMD patients was 0.829 (95%CI: 0.758–0.899, *P* < .01) for male and 0.812 (95%CI: 0.685–0.939, *P* < .01) for female, AUC for PhA to identify low SMI combined with low SMD patients was 0.801 (95%CI: 0.719–0.883, *P* < .01) for male and 0.773 (95%CI = 0.654–0.893, *P* < .01) for female, as shown in Table 4.

**Table 4:** The ability of PhA to identify low SMD, low SMI, and low SMI combined with low SMD.

PhA	Low SMI (n = 56)		Low SMD (n = 136)		Low SMI + low SMD (n = 51)	
	AUC	AUC	AUC	AUC	AUC	AUC
	Male	Female	Male	Female	Male	Female
	(95% CI)	(95% CI)	(95% CI)	(95% CI)	(95% CI)	(95%CI)
	0.772 (0.684–0.860)	0.784 (0.672–0.895)	0.829 (0.758–0.899)	0.812 (0.685–0.939)	0.801 (0.719–0.883)	0.773 (0.654–0.893)

95% CI = 95% confidence interval, AUC = area under curve, PhA = phase angle, SMI = skeletal muscle area index, SMD = skeletal muscle density.

## 4. Discussion

As a gold standard for measuring body composition, CT can simultaneously assess skeletal muscle quantity and quality. Meanwhile, increasing attention has been paid to the application of PhA in the assessment of human body composition.

The results of our study showed that PhA was lower in patients with low SMI, low SMD, and low SMI combined with low SMD. By analyzing the relationship between PhA and SMI and SMD, the results showed that PhA was positively associated with SMD and SMI, with correlation coefficients of 0.629 and 0.674, respectively. Logistic regression analysis showed that 1° reduction in PhA was associated with low SMI with an odds ratio of 4.331 and low SMD with an odds ratio of 6.418. Previous studies have also examined the relationship between PhA and SMI and SMD. The results of Looijaard et al showed that PhA was correlated with SMD and SMA, and the correlation coefficients were *R* = 0.701 and *R* = 0.542, respectively.^[24]^ The study of Souza NC et al^[18]^ in colorectal cancer patients showed that the correlation coefficients between PhA and SMD and SMI were *R* = 0.70 and *R* = 0.47, respectively, which were similar to the results of our study.

The results of this study demonstrated the ability of PhA to predict low SMI, low SMD, and low SMI combined with SMD. The AUC for PhA to identify low SMI patients was 0.772 for male and 0.784 for female; AUC for PhA to identify low SMD patients was 0.829 for male and 0.812 for female; AUC for PhA to identify low SMD combined with low SMD patients was 0.801 for male and 0.773 for female. This indicated that PhA can accurately predict patients with low SMI, low SMD, and low SMI combined with low SMD.

Based on the available evidence, the correlation between PhA and SMD can be explained as follows: with aging of the human body, a series of changes occur in the number and function of skeletal muscle mitochondria, including a decrease in the number, oxidative capacity, tricarboxylic acid cycle and *β*-oxidative capacity, resulting in fat infiltration in skeletal muscle cells. The increase in skeletal muscle fat infiltration may result in the transition of skeletal muscle fibers from type II to type I, a transition that results in impaired muscle contractility and decreased muscle strength, and fat tissue direct contact with muscle fibers may also cause damage to skeletal muscle cells and skeletal muscle function through lipotoxic proinflammatory cytokines.^[12,25,26]^ PhA is a marker of cellular health,^[27]^ which is also negatively associated with age. With aging, cell function and membrane integrity decline, mitochondrial function is impaired, and skeletal muscle fat infiltration occurs. Lower PhA means more severe skeletal muscle fat infiltration.

Low PhA is associated with low skeletal muscle mass and high fat mass.^[28]^ The decline in skeletal muscle mass occurs concurrently with an increase in skeletal muscle fat infiltration during aging. Loss of muscle mass can lead to a decrease in reactance, and an increase in fat mass can lead to an increase in resistance. Both the decrease in reactance and the increase in resistance were associated with a decrease in PhA.^[29]^ Thus, the reason for the correlation between PhA and SMI and SMD can be partially interpreted. Animal studies have also shown that raw impedance measurement parameters can reflect the actual condition of skeletal muscle tissue. The cross-sectional area of muscle fibers and intramuscular fat content in mice can be predicted by electrical impedance myography.^[30,31]^

The EWGSOP2’s revised recommendations emphasized the importance of the simultaneous assessment of skeletal muscle quantity and quality.^[32]^ The analysis in this study showed that the phase angle was highly related to SMI, which represents skeletal muscle quantity, and SMD, which can assess skeletal muscle quality, indicating that PhA can reflect the macroscopic quantity and microstructure and composition of skeletal muscle to a certain extent. PhA can effectively predict patients with low SMI and low SMD, indicating that the decrease in PhA may be a marker of decreased skeletal muscle quantity and quality.

Considering that skeletal muscle quantity and quality are both impaired in sarcopenia,^[32]^ so PhA may be used to diagnose sarcopenia directly. The study of Kilic MK et al exhibited that PhA can be used to diagnose sarcopenia.^[33]^ The study of Akamatsu Y et al also showed PhA is a good marker in diagnosing sarcopenia.^[34]^ In addition, Santana et al found that severe sarcopenia had lower PhA, which can be used as a marker of sarcopenia severity.^[35]^

Skeletal muscle mass derived from BIA relies on a specific regression equation and a constant hydration status of 73%. However, under conditions of hydration change such as edema or ascites, continuing to estimate muscle mass based on a constant hydration status will lead to an overestimation of skeletal muscle mass.^[36]^ PhA is a raw measurement of BIA that does not require the regression equations and does not need to assume constant hydration status. Thus, PhA may be more appropriate for the diagnose of sarcopenia in patients with a changing hydration status. A study by Yan Ding et al showed that PhA has a high predictive value for detecting sarcopenia in 346 patients undergoing maintenance hemodialysis.^[37]^ A study by Do JY et al found that PhA was independently associated with muscle mass, strength, and sarcopenia in patients undergoing peritoneal dialysis, and PhA could identify peritoneal dialysis patients at risk for sarcopenia.^[38]^

As for limitations, the single-center, retrospective, and observational design of the study prevented us from exploring the causal relationship between PhA and SMI and SMD. Prospective cohort studies with large samples are needed to validate our conclusions in the future.

Regarding strengths, there are no reports of such studies being conducted on Chinese hospitalized patients. As the raw measurements of BIA, PhA can avoid errors caused by the estimation formula and the assumed constant hydration state. In addition, the short time span between BIA and CT examinations avoided the influence of body composition changes.

In conclusion, PhA is an easily measurable tool and promising bioelectrical marker for evaluating skeletal muscle quantity and quality in hospitalized elderly patients. This study provides a reference for the widespread application of PhA. The use of PhA to evaluate skeletal muscle quantity and quality can help in better decision-making for elderly patients.

## Authors contributions

**Conceptualization:** Jiaxu Geng and Jingtong Wang.

**Data curation:** Yanan Wei.

**Formal analysis:** Jiaxu Geng.

**Investigation:** Qian Xue and Lihua Deng.

**Methodology:** Jiaxu Geng and Yanan Wei.

**Supervision:** Jingtong Wang.

**Validation:** Jingtong Wang.

**Writing – original draft:** Jiaxu Geng and Yanan Wei.

**Writing – review & editing:** Jiaxu Geng and Jingtong Wang.
